# Contemporary Views of Research Participant Willingness to Participate and Share Digital Data in Biomedical Research

**DOI:** 10.1001/jamanetworkopen.2019.15717

**Published:** 2019-11-20

**Authors:** Abhishek Pratap, Ryan Allred, Jaden Duffy, Donovan Rivera, Heather Sophia Lee, Brenna N. Renn, Patricia A. Areán

**Affiliations:** 1 Department of Biomedical Informatics and Medical Education, University of Washington, Seattle; 2 Sage Bionetworks, Seattle, Washington; 3 Department of Psychiatry & Behavioral Sciences, University of Washington, Seattle; 4 Department of Rehabilitation Medicine, University of Washington, Seattle; 5 Department of Family Medicine & Community Health, Rutgers Robert Wood Johnson Medical School, New Brunswick, New Jersey

## Abstract

**Importance:**

Using social media to recruit participants is a common and cost-effective practice. Willingness to participate (WTP) in biomedical research is a function of trust in the scientific team, which is closely tied to the source of funding and institutional connections.

**Objective:**

To determine whether WTP and willingness to share social media data are associated with the type of research team and online recruitment platform.

**Design, Setting, and Participants:**

This mixed-methods longitudinal survey and qualitative study was conducted over 2 points (T1 and T2) using Amazon’s Mechanical Turk (MTurk) platform. Participants were US adults aged 18 years or older who use at least 1 social media platform. Recruitment was stratified to match race/ethnicity proportions of the 2010 US Census. The volunteer sample consisted of 914 participants at T1, and 655 participants completed the follow-up survey 5 months later (T2).

**Main Outcomes and Measures:**

Outcomes were (1) past experience with online research and sharing social media data for research; (2) WTP in research advertised online; (3) WTP in a study sponsored by a pharmaceutical company, a university, or a federal agency; and (4) willingness to share social media data. Opinions were solicited regarding the European Union’s General Data Protection Regulation statute, which came into effect between T1 and T2.

**Results:**

Of 914 participants completing the first survey (T1), 604 (66.1%) were aged 18 to 39 years and 494 (54.0%) were female. Of these, 655 participants (71.7%) responded at T2. While 680 participants (74.4%) indicated WTP in biomedical research, only 454 (49.3%) were willing to share their social media data. Participants were significantly less likely to participate in federally sponsored (odds ratio [OR], 0.58; 95% CI, 0.51-0.64; *P* < .001) or pharmaceutical company (OR, 0.59; 95% CI, 0.53-0.66; *P* < .001) research than university-led studies. They were also less likely to share their social medial data for federal (OR, 0.65; 95% CI, 0.58-0.72; *P* < .001) or pharmaceutical company (OR, 0.50; 95% CI, 0.44-0.56; *P* < .001) research compared with academic studies. Willingness to participate in pharmaceutical company–led research decreased 11.89% from T1 to T2 (OR for T2, 0.62; 95% CI, 0.54-0.77; *P* < .001). Reasons for WTP were interest in furthering science, financial incentives, trust in the organization, and data security. While 63.0% of respondents reported seeing new privacy policy emails related to the General Data Protection Regulation law, only 27.1% indicated this positively influenced their WTP. Thematic analysis of responses indicated that WTP may improve with stronger data security measures.

**Conclusions and Relevance:**

This study suggests that researchers may see reduced online research participation and data sharing, particularly for research conducted outside academia.

## Key Points

**Question:**

Are people willing to participate in research advertised on the internet, and is willingness to participate associated with type of study sponsor?

**Findings:**

This mixed-methods survey and qualitative study of 914 respondents indicated that they were more likely to participate and share their social media data with researchers in university-led research studies than in studies conducted by the US federal government or pharmaceutical companies. However, only 49.3% indicated they would share their social media data at all.

**Meaning:**

These findings indicate that researchers may face challenges in recruiting representative samples when recruiting from internet platforms.

## Teaser

This mixed-methods survey and qualitative study investigates the association of willingness to participate in biomedical research and share social media data with source of study funding and online recruitment platform.

## Introduction

With 9 in 10 US adults seeking information on the web^1^ and 7 in 10 using social media platforms,^2^ the use of online mediums to recruit and to collect research data from diverse populations has become a common and cost-effective practice in health sciences research over the last 5 years.^3,4,5,6^ This form of recruitment and data collection is currently in use in large-scale biomedical research projects, such as the National Institute of Health’s Precision Medicine Initiative,^7^ which plans to recruit a diverse sample of 1 000 000 Americans through social media campaigns. Such projects also intend to collect digital information (electronic health records information, data from fitness devices, and even social media and web searches) to enhance our understanding of early risk factors for different disease states. Even social media companies are using digital data to inform better outcomes; for instance, Facebook has been able to use social media data to identify suicide risk in their users and, as a result, has formed a Compassion Team to address these issues.^8^

Recent data privacy violations^9,10,11^ potentially threaten the ability for biomedical researchers to recruit participants through online platforms and collect digital data from participants. Paramount to recruitment and subsequent participation in biomedical research is participant trust in science, the investigative team, and the management of personal information. Generations of biomedical research misconduct such as the Tuskegee syphilis experiment have influenced the public’s trust in biomedical research.^12^ A recent Pew Charitable Trust survey^13^ of trust in the internet found that even experts in digital security were mixed in their impressions that the general population will continue to share personal data online, with less than 50% of experts saying trust will improve with new regulations, and the remainder indicating that it will stay the same or erode over time. Another study^14^ from Australia found that while patients still feel that sharing personal information is important for biomedical research, there are considerable concerns voiced about how the data will be managed and that willingness to share such data is dependent on who is collecting the data. Lack of trust in studies advertised via the internet and social media and concerns about data security may bias samples collected in this manner.^15^ As a result, the use of these platforms for recruitment and data collection for biomedical research raises significant data privacy, ethics, ownership, and stewardship challenges^16^ for institutional review boards, researchers, and participants.

The purpose of this mixed-methods study was to ascertain (1) the general population’s willingness to participate (WTP) in biomedical research advertised on different digital platforms, (2) whether the study sponsor further modified the decision and WTP, (3) whether people are willing to share digital data in biomedical research, and (4) whether WTP improves in association with announcements regarding new data privacy laws.^17^

## Methods

### Recruitment and Eligibility

Participants were recruited using Amazon’s Mechanical Turk (MTurk),^18^ an online crowdsourcing platform where workers are paid to complete tasks such as data processing, problem-solving, and surveys. The platform is regularly used in health research^19^ and allows investigators to sample study participants from a larger, more representative, and more diverse population^20^ than typically seen in an in-person study at a fraction of the cost and time.

To be eligible, participants had to live in the United States, be aged 18 years or older, and use at least 1 social media platform. To ensure we were recruiting appropriate participants from the United States, we set the MTurk survey criteria to only include workers who lived and graduated high school in the United States (see eAppendix 1 in the Supplement for screening questions). The participant recruitment was stratified to match race/ethnicity proportions to that of the 2010 US Census data.^21^

### Procedures

**Figure 1.**
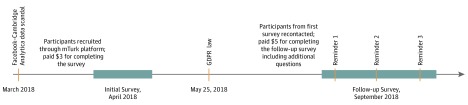
Overall Schematic of the Study Design The initial survey was deployed a month after the news about Facebook–Cambridge Analytica data privacy violations surfaced. The second survey was sent 5 months later in September 2018 to all the participants who responded to the first survey. A total of 3 reminders were sent to participants for completing the second survey. GDPR indicates General Data Protection Regulation; and mTurk, Amazon's Mechanical Turk.

The University of Washington institutional review board gave this study a category 2 exempt status because this is an opinion survey with participants the investigator cannot identify.^22^ Participants were provided with a brief explanation of the survey on the MTurk platform and were also informed that the team would contact them again in approximately 3 months to take a follow-up survey, which was also completely voluntary. Information about compensation was provided for T1 ($3) and T2 ($5) surveys. Once they consented, participants were asked to provide preliminary demographic information to determine eligibility. The MTurk platform was used to deploy the survey developed using REDCap (Research Electronic Data Capture)^23^ hosted at the Institute of Translational Health Sciences, University of Washington. REDCap is a secure web-based application developed through a multi-institutional collaborative effort and designed to support data capture for clinical and research studies. The first survey (T1) was administered in April 2018. The second survey (T2) was sent in September 2018 to all participants who completed the first survey. The primary goal of the T2 survey was to assess stability of WTP over time and to allow us to assess the association between WTP and the European Union’s General Data Protection Regulation (GDPR) law,^17^ which took effect on May 25, 2018. Participants were given up to 3 reminders to complete the second survey. Figure 1 gives an overview of the study procedures and eAppendix 2 and eAppendix 3 in the Supplement include the Screening, T1, and T2 surveys. This study followed the American Association for Public Opinion Research (AAPOR) reporting guideline.

#### Demographic Information

Demographic data (sex, race/ethnicity, age, and education) and social media use were self-reported by participants. Participants were also asked whether they had ever volunteered for an online study before and whether they had ever shared social media data for research purposes.

#### Survey Questions

The survey was developed by the authors and pilot tested to ensure clarity and understanding. The outcomes of interest were (1) participants’ past experience with online research, including whether they had ever shared social media data for research purposes; (2) WTP in biomedical research advertised on Google or Facebook; (3) WTP in a study sponsored by a pharmaceutical company (eg, Pfizer), a university (eg, University of California, Los Angeles), or a federal agency (eg, The National Institutes of Health); and (4) willingness to share social media data with a study sponsored by a pharmaceutical company, a university, or a federal agency. The T2 survey also included questions about the GDPR, which came into effect between the T1 and T2 surveys. Outcomes of interest were (1) whether participants had noticed emails from social media companies related to the GDPR law and (2) whether this new law reassured them about data security. For each question, participants were given an opportunity to explain the reason for their answers in an open field text box. See eAppendix 2 and eAppendix 3 in the Supplement for a copy of the T1 and T2 surveys.

### Statistical Analysis

Participants’ responses to structured survey questions were summarized using summary statistics. Differences in demographic characteristics between the participants who completed T1 and the subset who responded to T2 were assessed using a χ^2^ test. We have used a conservative minimum response rate based on AAPOR reporting guidelines^24^ to report the participant response rate for the T2 survey. Participant responses to the main outcomes of interest (WTP and willingness to share social media data) across the 2 survey points were evaluated using a logistic regression model based on generalized estimating equations.^25^ Briefly, the generalized estimating equation approach is a semiparametric method to estimate population-averaged effects by accounting for correlations in time-invariant data (that is, participant responses over time T1 and T2) using robust and unbiased standard errors. We also accounted for differences in participant responses due to demographic characteristics such as age, sex, and race/ethnicity. Due to small subgroup sample sizes, race/ethnicity was collapsed into a binary variable of minority or nonminority and participants within age groups 55 to 69 years and older than 70 years were collapsed into 1 age group of 55 years and older. To assess the stability of response over time, an interaction term indicating survey time (T1 vs T2) was included for each covariate in the generalized estimating equation model. We also assessed the combined association of the recruitment platform and study sponsors with WTP and data sharing using an interaction term. The significance (*P* values) of the model estimates were corrected for multiple testing using the false-discovery rate method. Two-tailed false-discovery rate–corrected *P* < .05 was considered statistically significant.

A mixed-methods approach combined quantitative and qualitative data with the function of expansion,^26^ allowing inductive qualitative data to provide the “why” to questions uncovered by the quantitative data. Missing data were not included in the analysis. Qualitative data were imported into Dedoose^27^ and analyzed using thematic analysis.^28^ Our research team consisted of investigators in digital mental health (A.P., P.A.A., and B.N.R.) and mixed-methods research (P.A.A.) and experts in the use of remote platforms for research recruitment (A.P. and P.A.A.). The team included an external qualitative methods consultant to verify coding and mitigate any potential conflicts of interest (H.S.L.). We developed the survey based on recent news events of social media data breaches and mishandling, with pragmatic interest in how such public discourse may influence participant recruitment and retention for studies. Two of us (D.R. and P.A.A.) independently familiarized themselves with the data and then coded a portion of survey responses to extract initial themes. Themes were developed and revised until saturation was reached. The themes were independently arrived at by the first 2 coders and then verified by another 2 of us (H.S.L. and R.A.). Data were iteratively reviewed (open coding) and collapsed to mutually exclusive themes (axial coding). For the second survey, we confirmed T1 themes, while still allowing for new themes to emerge. One of us (P.A.A.) reviewed and defined these new themes. Triangulation^29^ of quantitative and qualitative data allowed for convergence of themes and a more comprehensive understanding of WTP and willingness to share social media data. Illustrative quotes and themes are provided for a qualitative data audit trail. No power analysis was conducted, as this exploratory study did not attempt to demonstrate the effects of a particular magnitude and no similar standards of sample size exist for qualitative studies. Rather, we collected a sample large enough to contribute new knowledge to the analysis; during coding, saturation was achieved when no new themes emerged.^30^ All quantitative analysis was done using R statistical programming language (R Project for Statistical Computing).^31^

## Results

### Sample Characteristics

A total of 985 participants were recruited at T1. Of these, 655 participants (66.5% of the T1 responders) responded to the T2 survey. Responses from 71 participants (7.2%) were excluded from the data analysis owing to questionable data (eg, duplicate responses across questions, pasting of irrelevant text). No significant differences were seen in the participant demographic characteristics across the 2 surveys (Table 1). Overall, the cohort was relatively young, with 604 participants (66.1%) aged 18 to 39 years. The majority (67.3%) reported being non-Hispanic white, followed by Hispanic/Latino (13.9%) and African American (11.7%); 494 participants (54.0%) were female. Six hundred fifty-eight participants (72%) indicated that they had participated in online research previously, with 151 of this subsample (23%) stating they had shared social media data for research purposes.

**Table 1.** Comparison of Participant Demographic Characteristics Across the 2 Surveys

Characteristic	No. (%)		*P* Value
	T1: April 2018 (n = 914)	T2: September 2018 (n = 655)	
Age, y			
18-24	76 (8.3)	51 (7.8)	.97
25-39	528 (57.8)	379 (57.9)	
40-54	226 (24.7)	167 (25.5)	
55-69	72 (7.9)	48 (7.3)	
≥70	12 (1.3)	10 (1.5)	
Female	494 (54.0)	346 (52.8.)	.67
Race/ethnicity			
White	615 (67.3)	439 (67.0)	.84
Hispanic/Latino	127 (13.9)	82 (12.5)	
Black/African American	107 (11.7)	86 (13.1)	
Asian	52 (5.7)	40 (6.1)	
Hawaiian/Pacific Islander/Native American/Alaska Native	13 (1.4)	8 (1.2)	

Abbreviations: T1, first survey; T2, second survey.

### T1 Analyses

#### WTP by Recruitment Platform and Sponsor

We identified significant differences in WTP in research by recruitment platform and by the study sponsor. Of all T1 respondents, 680 (74.4%) indicated WTP in a biomedical research study run by 1 of the 3 institutions (either a university, a federal agency, or a pharmaceutical company). Compared with a study sponsored by a university, participants were less likely to report WTP in a study sponsored by a federal agency (odds ratio [OR], 0.58; 95% CI, 0.51-0.64; *P* < .001) or a pharmaceutical company (OR, 0.59; 95% CI, 0.53-0.66; *P* < .001). The WTP was also significantly lower for older participants (OR for those aged 55 years and older, 0.36; 95% CI, 0.22-0.61; *P* < .001) compared with adults aged 18 to 24 years. Willingness to participate was also significantly greater for recruitment through Google compared with Facebook advertisements (OR, 1.24; 95% CI, 1.10-1.41; *P* < .001; university sponsored: 61.6% vs 56.5%; federal agency led: 49.5% vs 43.5%; and pharmaceutical led: 47.9% vs 42.9%, respectively). No significant differences in WTP were observed by participant sex or race/ethnicity (Table 2).

**Table 2.** Willingness to Participate in Online Biomedical Research at Time T1 and Change Over Time T2 (Interaction Effect)

Characteristic	Odds Ratio (95% CI)^a^	
	T1 Survey	Change Over Time (Interaction Effect With T2 Survey)
Intercept	2.03 (1.40-2.95)^b^	
Survey T2	0.78 (0.45-1.35)	
Sponsor		
University	1 [Reference]	
Pharmaceutical	0.58 (0.51-0.64)^b^	0.62 (0.54-0.77)^b^
Federal	0.59 (0.53-0.66)^b^	0.84 (0.71-1.00)
Platform		
Facebook	1 [Reference]	
Google	1.24 (1.10-1.41)^b^	0.77 (0.64-0.92)^b^
Age, y		
18-24	1 [Reference]	
25-39	0.62 (0.43-0.89)^c^	1.47 (0.86-2.49)
40-54	0.54 (0.36-0.81)^c^	1.93 (1.09-3.41)
≥55	0.36 (0.22-0.61)^b^	2.95 (1.46-5.92)^b^
Sex		
Female	1 [Reference]	
Male	0.97 (0.79-1.20)	0.94 (0.73-1.22)
Race		
Racial/ethnic minority	1 [Reference]	
White	1.14 (0.91-1.42)	0.88 (0.66-1.16)
Sponsor and platform		
Pharmaceutical and Google	0.99 (0.88-1.12)	0.93 (0.77-1.13)
Federal and Google	1.03 (0.91-1.15)	1.11 (0.94-1.32)

Abbreviations: T1, first survey; T2, second survey.

^a^ Odds ratios were determined using logistic regression based on the method of generalized estimating equations including assessing the association of participants’ demographic characteristics, study sponsor, and recruitment platform with willingness to participate.

^b^ Statistically significant at false discovery rate–corrected *P* < .001.

^c^ Statistically significant at false discovery rate–corrected *P* < .05.

Common themes derived from our qualitative analysis found that respondents were willing to participate based on (1) altruistic reasons, (2) financial incentives, and (3) trust or credibility of the sponsor*.* Themes regarding disincentive to participate were concerns about data security and lack of trust in the study sponsor. See eTable 3 in the Supplement for illustrative participant quotes that represent these themes.

#### Willingness to Share Social Media Data

Most participants (464 [50.8%]) preferred not to share their social media data with any entity. The remaining 454 participants (49.3%) were willing to share their data with at least 1 of the 3 study sponsors. Of those willing to share, 219 (23.9%) were willing to share with all 3, 120 (13.1%) with 2 of the 3 sponsors, and the remaining 111 (12.1%) with only 1 institution. Participants were significantly more likely to share their social media data in university-led research (45.0% of the respondents) compared with research sponsored by a federal agency (35.2% of the respondents; OR, 0.65; 95% CI, 0.58-0.72; *P* < .001) or pharmaceutical company–sponsored research (29.5% of the respondents; OR, 0.50; 95% CI, 0.44-0.56; *P* < .001). Willingness to share social media data was also lower for participants aged 40 to 54 years (OR, 0.46; 95% CI, 0.28-0.74; *P* < .001) and those aged 55 years and older (OR, 0.37; 95% CI, 0.20-0.69; *P* < .001) compared with adults aged 18 to 39 years. No significant difference in willingness to share by race/ethnicity or sex was observed (Table 3). Major themes were similar to themes for WTP, with universities being seen as trustworthy and participants questioning the trustworthiness of pharmaceutical and federal sponsors. See eTable 4 in the Supplement for illustrative participant quotes that represent these themes.

**Table 3.** Willingness to Share Social Media Data in Online Biomedical Research at Time T1 and Change Over Time T2 (Interaction Effect)

Characteristic	Odds Ratio (95% CI)^a^	
	T1 Survey	Change Over Time (Interaction Effect With T2 Survey)
Intercept	1.59 (1.03-2.47)	
Survey T2	0.81 (0.47-1.39)	
Sponsor		
University	1 [Reference]	
Federal	0.65 (0.58-0.72)^b^	0.79 (0.67-0.93)^c^
Pharmaceutical	0.50 (0.44-0.56)^b^	0.76 (0.65-0.93)^c^
Age group, y		
18-24	1 [Reference]	
25-39	0.61 (0.40-0.94)	1.19 (0.67-2.10)
40-54	0.46 (0.28-0.74)^b^	1.58 (0.86-2.92)
≥55	0.37 (0.20-0.69)^b^	1.58 (0.71-3.52)
Sex		
Female	1 [Reference]	
Male	0.98 (0.77-1.25)	0.82 (0.60-1.12)
Race		
Racial/ethnic minority	1 [Reference]	
White	0.91 (0.70-1.18)	0.94 (0.67-1.32)

^a^ Odds ratios were determined using logistic regression based on the method of generalized estimating equations including assessing the association of participants’ demographic characteristics, study sponsor, and recruitment platform with willingness to share social media data.

^b^ Statistically significant at false discovery rate–corrected *P* < .001.

^c^ Statistically significant at false discovery rate–corrected *P* < .05.

### T2 Analysis

#### WTP by Recruitment Platform and Sponsor

**Figure 2.**
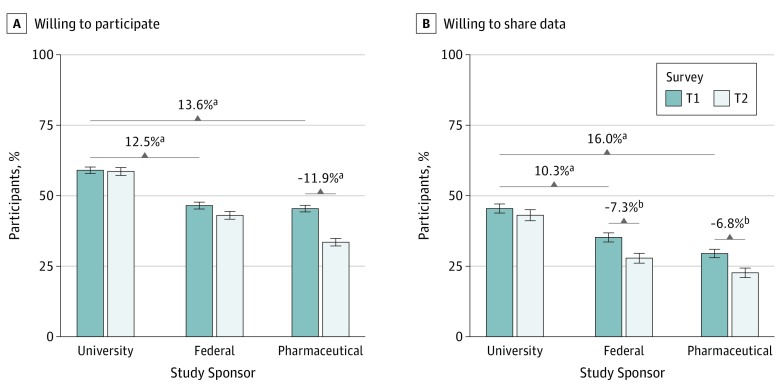
Proportion of Participants Willing to Participate and Share Their Social Media Data Proportions of participants willing to participate in biomedical research (A) and share their social media data for biomedical research (B) in the first survey (T1) and the second survey (T2). Error bars indicate bootstrapped estimates of variations in participants' responses (1 SD). ^a^Statistically significant at false discovery rate–corrected *P* < .001. ^b^Statistically significant at false discovery rate–corrected *P* < .05.

Of 914 T1 participants, 655 (66.5% of the respondents for T1) responded to the T2 survey. Willingness to participate only changed for pharmaceutical-sponsored research, which decreased 11.89% by T2 (OR for T2 compared with T1, 0.62; 95% CI, 0.54-0.77; *P* < .001) (Figure 2A). Older participants (≥55 years) who responded at T2 showed significantly greater WTP (OR for T2 compared with T1, 2.95; 95% CI, 1.46-5.92; *P* < .001) compared with adults aged 18 to 39 years (Table 2). Participant preference for recruitment via Google advertisements as observed in T1 (OR, 1.24) also decreased over time (decrease in OR, 0.77; 95% CI, 0.64-0.92; *P* < .001) and was nearly the same as the Facebook platform (OR for Google vs Facebook at T2, 0.96; 95% CI, 0.70-1.38) (see eTable 1 and eTable 2 in the Supplement for further breakdown of groupwise proportions). No new themes emerged between T2 and T1 regarding WTP.

#### Willingness to Share Social Media Data

Willingness to share social media data decreased significantly for all but university-led studies. While 43.1% of T2 respondents were willing to share social media with university-led studies, willingness to share with pharmaceutical companies decreased 6.84% to 29.5% (OR, 0.50; 95% CI, 0.44-0.56; *P* < .001) and decreased 7.32% to 35.3% with federally led research studies (OR, 0.65; 95% CI, 0.58-0.72; *P* < .001) (Figure 2B). Continued privacy and data security concerns reported in the news were noted as a problem in the qualitative data.

#### European GDPR Law

Four hundred thirteen participants (63.0%) reported seeing GDPR-related emails and/or advertisements by T2. No significant difference in WTP or willingness to share social media data was found between participants who reported seeing the GDPR-related emails and those who did not. 112 participants (27.1%) said that the GDPR-related messages made them feel more secure about their data and provided proof that the organization was working on its data security. As 1 respondent explained, “It shows me that they make notice of our concerns and are fixing them.” However, 301 participants (72.9%) felt the GDPR-related messages did not regain their trust. As 1 respondent stated, “I think the ads are just aimed at fixing a public relations problem. They still make their money from collecting our data and selling it and they aren’t going to stop.”

## Discussion

This mixed-methods study found that trust in the use of digital platforms, such as Google search and Facebook, was associated with participants’ WTP in and share social media data with biomedical research efforts. Moreover, trust in research entities was low, with most participants indicating an unwillingness to share social media data with federally sponsored or pharmaceutical company–led research. Although participants acknowledged the importance of participating in biomedical research and indicated they would do so for altruistic reasons, concerns about privacy and misuse of their personal data appeared to outweigh the perceived importance of volunteering to participate in such research.^32^ Issues of data security and mistrust may adversely affect research projects that plan to rely on large-scale recruitment through digital platforms. Recruitment of this nature, without a concerted effort to address participant mistrust of how their data will be managed, may result in the recruitment of large but biased samples that are not representative of the intended population. This could have a significant impact on the generalizability of outcomes from biomedical research.

Although our thematic analysis indicated that better data security measures and transparency of data use may mitigate concerns regarding participation, less than a quarter of our sample indicated that they were reassured by recent attempts at regulation such as the GDPR policies. The findings from this study are understandable in light of growing evidence that data privacy policies available on digital platforms do not accurately disclose how that information is used. One recent article^33^ found that many health apps share digital data with companies like Facebook and Google but fail to disclose this in their data privacy policies. A qualitative study^32^ of participants’ willingness to share research data reported similar findings, with trust in the research team and fears related to misuse arising as major concerns by potential participants. Our findings, combined with others, suggest that social media campaigns and policies to address how privacy and data security will be improved may not be sufficient to address WTP in online research and share digital data. As our survey results showed, participants remained mistrustful of these platforms several months after the platforms had sent out messages addressing their data security problems. However, partnership with universities and other trusted entities to develop better policies may be a useful solution, given how consistently our participants expressed trust in university-led research. As a number of studies indicate, participants’ trust in research is closely linked to the institution conducting the research.^34^

### Limitations

The findings from this survey would benefit from further research and should be viewed with the following limitations in mind. First, this is a general population survey of participant impressions about WTP in research and willingness to share personal data. To confirm our findings, a study specifically comparing recruitment avenues would need to be conducted. Second, participants were identified through MTurk, and therefore the representativeness of the findings may be influenced by our sample selection method, even though participants were recruited to match the race/ethnicity of the 2010 US Census data.^21^ Although MTurk participants are likely to be more aware of data sharing policies and more comfortable with online research than the general public, recent studies suggest that for research of this nature, these samples tend to be as good as, or better than, in-person surveys.^35^ Third, our sample was not recruited specifically to test hypotheses about racial/ethnic, sex, or age differences. Particularly in regard to our findings that WTP seemed to improve in older populations over time, we believe this to be an artifact of the small sample of older adults who participated in this survey, and that in all likelihood these are not participants who are representative of older adults in the general population. Thus, to truly understand demographic differences in WTP, a large study that oversamples participants from different demographic groups will need to be conducted. In addition, the phrasing of survey questions listing depression as a health condition could also have negatively affected study participants’ WTP, given the stigma associated with the disorder.^36^ Despite these limitations, the data are still useful for both informing recruitment practices and providing information about the concerns people have regarding the secure management of social media data for research purposes, particularly at this time.

## Conclusions

In conclusion, WTP in biomedical research advertised on social media platforms and search engines, as well as the willingness to share digital data with researchers, have been affected by recent news on the misuse of such data. Although university-led research is seen as more trustworthy than federally led or pharmaceutical company–led research, WTP is still affected. Despite these concerns, social media provides opportunities for conducting biomedical research at scale,^37^ including enrolling minority populations,^5^ and could help improve diversity in clinical trials, many of which are discontinued early due to recruitment challenges.^38^ It will be important for researchers and research organizations to work more closely with participant communities to address concerns about data sharing and privacy.

## Notes

Supplement. **eAppendix 1.** Screening Questions **eAppendix 2.** T1 Survey **eAppendix 3.** T2 Survey **eTable 1.** Number and Proportion of Participants Willing to Participate Seeing a Study Ad as Part of the Google Search Results **eTable 2. **Number and Proportion of Participants Willing to Share Social Media Data Seeing a Study Ad as Part of the Google Search Results **eTable 3.** Reasons for Agreeing or Declining to Participate in Research Studies Advertised Online **eTable 4.** Reasons for Agreeing or Declining to Share Social Media Data for Research Studies
