# Supplementary material for: Contemporary Views of Research Participant Willingness to Participate and Share Digital Data in Biomedical Research
Source: JAMA Netw Open. 2019 Nov 20;2(11):e1915717. doi: 10.1001/jamanetworkopen.2019.15717 (PMC6902809; doi:10.1001/jamanetworkopen.2019.15717)
Supplement: Supplement. — eAppendix 1. Screening Questions eAppendix 2. T1 Survey eAppendix 3. T2 Survey eTable 1. Number and Proportion of Participants Willing to Participate Seeing a Study Ad as Part of the Google Search Results eTable 2. Number and Proportion of Participants Willing to Share Social Media Data Seeing a Study Ad as Part of the Google Search Results eTable 3. Reasons for Agreeing or Declining to Participate in Research Studies Advertised Online eTable 4. Reasons for Agreeing or Declining to Share Social Media Data for Research Studies [file jamanetwopen-2-e1915717-s001.pdf]

Supplementary Online Content

Pratap A, Allred R, Duffy J, et al. Contemporary views of research participant willingness to participate and share digital data in biomedical research. *JAMA Netw Open*. 2019;2(11):e1915717. doi:10.1001/jamanetworkopen.2019.15717

eAppendix 1. Screening Questions
eAppendix 2. T1 Survey
eAppendix 3. T2 Survey
eTable 1. Number and Proportion of Participants Willing to Participate Seeing a Study Ad as Part of the Google Search Results
eTable 2. Number and Proportion of Participants Willing to Share Social Media Data Seeing a Study Ad as Part of the Google Search Results
eTable 3. Reasons for Agreeing or Declining to Participate in Research Studies Advertised Online
eTable 4. Reasons for Agreeing or Declining to Share Social Media Data for Research Studies

This supplementary material has been provided by the authors to give readers additional information about their work.

eAppendix 1. Screening Questions

1. Do you use social media?

- ◯ Yes
- ◯ No

1a. If yes, check all that apply:

- ☐ a. Facebook
- ☐ b. Snapchat
- ☐ c. Instagram
- ☐ d. Twitter
- ☐ e. Pinterest
- ☐ f. LinkedIn
- ☐ g. Tumblr
- ☐ h. Vero
- ☐ i. Other:

1b. If Other, please indicate:

---

2. How old are you?

- ◯ Under 18
- ◯ 18-24
- ◯ 25-39
- ◯ 40-54
- ◯ 55-69
- ◯ 70+

---

3. Do you live in the United States?

- ◯ Yes
- ◯ No

---

4. Did you graduate from high school in the United States?

- ◯ Yes
- ◯ No

---

5. What is your race and/or ethnicity? Check all that apply.

- ☐ White / Caucasian
- ☐ Black / African American
- ☐ Asian
- ☐ Hawaiian or Pacific Islander, Native American
- ☐ Hispanic or Latino/a

---

6. What is your gender?

- ◯ Male
- ◯ Female
- ◯ Non-Binary

© 2019 Pratap A et al. *JAMA Network Open.*

eAppendix 2. T1 Survey

1. Have you ever intentionally shared your social media or search data with a research team before?

- ◯ Yes
- ◯ No

2. Have you ever volunteered for a research study online before?

- ◯ Yes
- ◯ No

3. If you saw an ad for a pharmaceutical company sponsored research study for a health condition such as depression (for example, Pfizer for Prozac) on Facebook, would you be willing to participate?

- ◯ Yes
- ◯ No

3a. Tell us more about the reason(s) behind your answer.

4. If you saw an ad for a pharmaceutical company sponsored research study for a health condition such as depression (for example, Pfizer for Prozac) during a Google search, would you be willing to participate?

- ◯ Yes
- ◯ No

4a. Tell us more about the reason(s) behind your answer.

5. If you saw an ad for a university sponsored research study for a health condition such as depression (for example, UCLA for Prozac) on Facebook, would you be willing to participate?

- ◯ Yes
- ◯ No

5a. Tell us more about the reason(s) behind your answer.

© 2019 Pratap A et al. *JAMA Network Open.*

6. If you saw an ad for a university sponsored research study for a health condition such as depression (for example, UCLA for Prozac) during a Google search, would you be willing to participate?

○ Yes
○ No

6a. Tell us more about the reason(s) behind your answer.

7. If you saw an ad for a federally sponsored research study for a health condition such as depression (for example, the National Institutes of Health for Prozac) on Facebook, would you be willing to participate?

○ Yes
○ No

7a. Tell us more about the reason(s) behind your answer.

8. If you saw an ad for a federally sponsored research study for a health condition such as depression (for example, the National Institutes of Health for Prozac) during a Google Search, would you be willing toparticipate?

○ Yes
○ No

8a. Tell us more about the reason(s) behind your answer.

9. Would you be willing to share your social media data (Facebook, Twitter, Instagram posts) with a pharmaceutical company sponsored research study for a health condition like depression (for example, Pfizer)?

○ Yes
○ No

9a. Tell us more about the reason(s) behind your answer.

10. Would you be willing to share your social media data (Facebook, Twitter, Instagram posts) with a university sponsored research study for a health condition like depression (for example, the University of California)?

○ Yes
○ No

10a. Tell us more about the reason(s) behind your answer.

© 2019 Pratap A et al. *JAMA Network Open.*

11. Would you be willing to share your social media data (Facebook, Twitter, Instagram posts) with a federally sponsored research study for a health condition like depression (for example, the National Institutes of Health)?

- ◯ Yes
- ◯ No

11a. Tell us more about the reason(s) behind your answer.

12. Would you be willing to share your social media data (Facebook, Twitter, Instagram posts) with a health plan sponsored research study for a health condition like depression (for example, United Health Care)?

- ◯ Yes
- ◯ No

12a. Tell us more about the reason(s) behind your answer.

13. Would you be willing to share your social media data (Facebook, Twitter, Instagram posts) with a foundation sponsored research study for health condition like depression (for example, the Bill & Melinda Gates Foundation)?

- ◯ Yes
- ◯ No

13a. Tell us more about the reason(s) behind your answer.

© 2019 Pratap A et al. *JAMA Network Open.*

eAppendix 3. T2 Survey

1. Since the last survey, have you stopped using any particular social media?

- ◯ Yes
- ◯ No

---

a. If yes, check all that apply:

- ☐ a. Facebook
- ☐ b. Snapchat
- ☐ c. Instagram
- ☐ d. Twitter
- ☐ e. Pinterest
- ☐ f. LinkedIn
- ☐ g. Tumblr
- ☐ h. Vero
- ☐ i. Reddit
- ☐ j. Other:

If Other, please indicate:

---

2. Since the last survey, have you joined any particular social media platforms?

- ◯ Yes
- ◯ No

---

a. If yes, check all that apply:

- ☐ a. Facebook
- ☐ b. Snapchat
- ☐ c. Instagram
- ☐ d. Twitter
- ☐ e. Pinterest
- ☐ f. LinkedIn
- ☐ g. Tumblr
- ☐ h. Vero
- ☐ i. Reddit
- ☐ j. Other:

If Other, please indicate:

---

3. Have you ever intentionally shared your social media or search data with a research team before?

- ◯ Yes
- ◯ No

© 2019 Pratap A et al. *JAMA Network Open.*

a. If yes, check all that apply:

☐ a. Facebook
☐ b. Snapchat
☐ c. Instagram
☐ d. Twitter
☐ e. Pinterest
☐ f. LinkedIn
☐ g. Tumblr
☐ h. Vero
☐ i. Reddit
☐ j. Other:

If Other, please indicate:

a. If no, what kind of social media data would you be willing to share with a research team?

☐ a. Facebook
☐ b. Snapchat
☐ c. Instagram
☐ d. Twitter
☐ e. Pinterest
☐ f. LinkedIn
☐ g. Tumblr
☐ h. Vero
☐ i. Reddit
☐ j. Other:
☐ k. I would not share any social media data with a research team.

If Other, please indicate:

4. Have you ever volunteered for a research study online before?

◯ Yes
◯ No

a. What category below best describes the research study you participated in? (Check all that apply.)

☐ a. mTurk-based studies
☐ b. Online health surveys other than mTurk
☐ c. Online focus groups other than mTurk
☐ d. Online marketing surveys other than mTurk
☐ e. Other online surveys
☐ f. University Sponsored research
☐ g. Pharma Sponsored research
☐ h. Non-profit Sponsored research
☐ i. Other:

If Other, please indicate:

b. Please tell us briefly why you volunteered to participate in that online research study.

© 2019 Pratap A et al. *JAMA Network Open.*

a. If no, please tell us more about the reason behind your answer.

5. Would you be willing to click an ad to learn more about a research study for a health condition?

- ◯ Yes
- ◯ No

a. If yes, on which social media sites would you be most willing to interact with an ad?

- ☐ a. Facebook
- ☐ b. Snapchat
- ☐ c. Instagram
- ☐ d. Twitter
- ☐ e. Pinterest
- ☐ f. LinkedIn
- ☐ g. Tumblr
- ☐ h. Vero
- ☐ i. Reddit
- ☐ j. Other:

If Other, please indicate:

6. Would you be more willing to click an ad to learn more (not necessarily participate) about an online research study for a health condition based on who is sponsoring the study?

- ◯ Yes
- ◯ No

a. If yes, which would you be most willing to click on? A study sponsored by:

- ◯ University (e.g: University of California, Eckerds College)
- ◯ Non-profit (e.g: Bill & Melinda Gates Foundation)
- ◯ Federal Agency (e.g: National Institutes of Health)
- ◯ Pharmaceutical company (e.g: Novartis, Pfizer)

7. If you saw an ad for a pharmaceutical company sponsored research study for a health condition such as depression (for example, from Pfizer for Prozac) on Facebook, would you be willing to participate?

- ◯ Yes
- ◯ No

a. Tell us more about the reason behind your answer.

8. If you saw an ad for a pharmaceutical company sponsored research study for a health condition such as depression (for example, from Pfizer for Prozac) during a Google search, would you be willing to participate?

- ◯ Yes
- ◯ No

© 2019 Pratap A et al. *JAMA Network Open.*

a. Tell us more about the reason behind your answer.

9. If you saw an ad for a university sponsored research study for a health condition such as depression (for example, UCLA for Prozac) on Facebook, would you be willing to participate?

- ◯ Yes
- ◯ No

a. Tell us more about the reason behind your answer.

b. What if the study was sponsored by a lesser known university (e.g.: Eckerds College in Florida)?

- ◯ Yes
- ◯ No

c. Tell us more about the reason behind your answer.

10. If you saw an ad for a university sponsored research study for a health condition such as depression (for example, UCLA for Prozac) during a Google search, would you be willing to participate?

- ◯ Yes
- ◯ No

a. Tell us more about the reason behind your answer.

11. If you saw an ad for a federally sponsored research study for a health condition such as depression (for example, the National Institutes of Health for Prozac) on Facebook, would you be willing to participate?

- ◯ Yes
- ◯ No

a. Tell us more about the reason behind your answer.

12. If you saw an ad for a federally sponsored research study for a health condition such as depression (for example, the National Institutes of Health for Prozac) during a Google search, would you be willing to participate?

- ◯ Yes
- ◯ No

© 2019 Pratap A et al. *JAMA Network Open.*

a. Tell us more about the reason behind your answer.

13. Would you be willing to share your social media data (Facebook, Twitter, Instagram posts) with a pharmaceutical company sponsored research study for a health condition like depression (for example, Pfizer)?

- ○ Yes
- ○ No

a. Tell us more about the reason behind your answer.

14. Would you be willing to share your social media data (Facebook, Twitter, Instagram posts) with a university sponsored research study for a health condition like depression (for example, the University of California)?

- ○ Yes
- ○ No

a. Tell us more about the reason behind your answer.

15. Would you be willing to share your social media data (Facebook, Twitter, Instagram posts) with a federally sponsored research study for a health condition like depression (for example, the National Institutes of Health)?

- ○ Yes
- ○ No

a. Tell us more about the reason behind your answer

16. Would you be willing to share your social media data (Facebook, Twitter, Instagram posts) with a health plan sponsored research study for a health condition like depression (for example, United Health Care)?

- ○ Yes
- ○ No

a. Tell us more about the reason behind your answer.

© 2019 Pratap A et al. *JAMA Network Open.*

17. Would you be willing to share your social media data (Facebook, Twitter, Instagram posts) with an foundation sponsored research study for a health condition like depression (for example, Bill & Melinda Gates Foundation)?

◯ Yes
◯ No

a. Tell us more about the reason behind your answer.

18. Based on the results of our first survey, we noticed a higher percentage of users are willing to participate in University-sponsored research studies recruiting through Facebook ads compared to Google ads.

a. Are you more willing to participate in a university-sponsored research study you found out about from an ad on Facebook as opposed to one you saw on Google?

◯ Yes
◯ No

b. Why? Why not?

Recently, social media companies have been more open about their privacy policies and their commitment to your privacy especially in the wake of new the European General Data Protection Regulation (GDPR)(https://www.eugdpr.org/). For example, Facebook has recently released a video. Additionally, other companies sent out emails highlighting changes in their data usage and privacy policies.

19. Have you seen any data privacy and security emails and/or advertisements from Facebook, Google, or other social media platforms in the last 3 months?

◯ Yes
◯ No

a. Having seen the ad, do you feel more secure and trust the companies with your social media data, its usage and privacy?

◯ Yes
◯ No

b. Finally, having seen the new ads and/or emails about data sharing and privacy policies, has your opinion on trusting online research advertisements on Facebook and Google changed?

◯ Yes
◯ No

c. Why or why not?

Any additional comments?

© 2019 Pratap A et al. *JAMA Network Open.*

eTable 1: Number and proportion of participants willing to participate seeing a study ad as part of the Google Search results

Recruitment Platform Facebook	T1 Survey						T2 Survey					
	Pharma		Federal		University		Pharma		Federal		University	
Willingness to Participate	Yes	No	Yes	No	Yes	No	Yes	No	Yes	No	Yes	No
N(%)	392 (42.9)	522 (57.1)	398 (43.5)	516 (56.5)	516 (56.5)	398 (43.5)	228 (34.9)	424 (65.0)	274 (42.1)	378 (57.9)	386 (59.2)	266 (40.8)
**Age (%)**												
18-24	38 (9.7)	38 (7.3)	41 (10.3)	35 (6.8)	48 (9.3)	28 (7.0)	21 (9.2)	30 (7.1)	22 (8.0)	29 (7.7)	28 (7.3)	23 (8.6)
25-39	237 (60.5)	291 (55.7)	234 (58.8)	294 (57.0)	315 (61.0)	213 (53.5)	119 (52.2)	259 (61.1)	161 (58.8)	217 (57.4)	220 (57.0)	158 (59.4)
40-54	96 (24.5)	130 (24.9)	93 (23.4)	133 (25.8)	121 (23.4)	105 (26.4)	69 (30.3)	96 (22.6)	64 (23.4)	101 (26.7)	102 (26.4)	63 (23.7)
55 and over	21 (5.4)	63 (12.1)	30 (7.5)	54 (10.4)	32 (6.2)	52 (13.1)	19 (8.4)	39 (9.2)	27 (9.9)	31 (8.2)	36 (9.3)	22 (8.2)
**Gender**												
Male (%)	174 (44.4)	246 (47.1)	191 (48.0)	229 (44.4)	244 (47.3)	176 (44.2)	97 (42.5)	211 (49.8)	130 (47.4)	178 (47.1)	177 (45.9)	131 (49.2)
**Race/Ethnicity (%)**												
White	260 (66.3)	355 (68.0)	271 (68.1)	344 (66.7)	353 (68.4)	262 (65.8)	149 (65.4)	288 (67.9)	184 (67.2)	253 (66.9)	266 (68.9)	171 (64.3)
Asian	21 (5.4)	31 (5.9)	22 (5.5)	30 (5.8)	28 (5.4)	24 (6.0)	13 (5.7)	27 (6.4)	16 (5.8)	24 (6.3)	20 (5.2)	20 (7.5)
Black/African American	47 (12.0)	60 (11.5)	46 (11.6)	61 (11.8)	57 (11.0)	50 (12.6)	36 (15.8)	50 (11.8)	36 (13.1)	50 (13.2)	48 (12.4)	38 (14.3)
Hawaiian/PI/ NAmerican/ Alaska N	9 (2.3)	4 (0.8)	6 (1.5)	7 (1.4)	8 (1.6)	5 (1.3)	3 (1.3)	5 (1.2)	1 (0.4)	7 (1.9)	2 (0.5)	6 (2.3)
Hispanic/Latino	55 (14.0)	72 (13.8)	53 (13.3)	74 (14.3)	70 (13.6)	57 (14.3)	27 (11.8)	54 (12.7)	37 (13.5)	44 (11.6)	50 (13.0)	31 (11.7)

© 2019 Pratap A et al. *JAMA Network Open.*

eTable 2: Number and proportion of participants willing to share social media data seeing a study ad as part of the Google Search results

Recruitment Platform Google	T1 Survey						T2 Survey					
	Pharma		Federal		University		Pharma		Federal		University	
**Willingness to Participate**	Yes	No	Yes	No	Yes	No	Yes	No	Yes	No	Yes	No
N(%)	438 (47.9)	476 (52.1)	452 (49.5)	462 (50.5)	563 (61.6)	351 (38.4)	209 (32.1)	443 (67.9)	287 (44)	365 (56)	378 (57.9)	274 (42.1)
**Age (%)**												
18-24	48 (11.0)	28 (5.9)	48 (10.6)	28 (6.1)	59 (10.5)	17 (4.8)	18 (8.6)	33 (7.4)	23 (8.0)	28 (7.7)	28 (7.4)	23 (8.4)
25-39	248 (56.6)	280 (58.8)	263 (58.2)	265 (57.4)	327 (58.1)	201 (57.3)	113 (54.1)	265 (59.8)	164 (57.1)	214 (58.6)	212 (56.1)	166 (60.6)
40-54	111 (25.3)	115 (24.2)	102 (22.6)	124 (26.8)	132 (23.4)	94 (26.8)	59 (28.2)	106 (23.9)	72 (25.1)	93 (25.5)	100 (26.5)	65 (23.7)
55 and over	31 (7.1)	53 (11.2)	39 (7.1)	45 (9.7)	45 (8.0)	39 (11.1)	19 (9.1)	39 (8.8)	28 (9.8)	30 (8.2)	38 (10)	20 7.3)
**Gender**												
Male (%)	193 (44.1)	227 (47.7)	208 (46.0)	212 (45.9)	268 (47.6)	152 (43.3)	90 (43.1)	218 (49.2)	138 (48.1)	170 (46.6)	176 (46.6)	132 (48.2)
**Race/Ethnicity (%)**												
White/Caucasian	287 (65.5)	328 (68.9)	306 (67.7)	309 (66.9)	384 (68.2)	231 (65.8)	129 (61.7)	308 (69.5)	192 (66.9)	245 (67.1)	264 (69.8)	173 (63.1)
Asian	20 (4.6)	32 (6.7)	22 (4.9)	30 (6.5)	27 (4.8)	25 (7.1)	11 (5.3)	29 (6.5)	17 (5.9)	23 (6.3)	18 (4.8)	22 (8.0)
Black/African American	59 (13.5)	48 (10.1)	53 (11.7)	54 (11.7)	66 (11.7)	41 (11.7)	36 (17.2)	50 (11.3)	39 (13.6)	47 (12.9)	50 (13.2)	36 (13.1)
Hawaiian/PI/ NAmerican/Alaska N	8 (1.8)	5 (1.1)	6 (1.3)	7 (1.5)	9 (1.6)	4 (1.1)	3 (1.4)	5 (1.1)	3 (1.0)	5 (1.4)	2 (0.5)	6 (2.2)
Hispanic/Latino	64 (14.6)	63 (13.2)	65 (14.4)	62 (13.4)	77 (13.7)	50 (14.2)	30 (14.4)	51 (11.5)	36 (12.5)	45 (12.3)	44 (11.6)	37 (13.5)

© 2019 Pratap A et al. *JAMA Network Open.*

eTable 3: Reasons for agreeing or declining to participate in research studies advertised online.

	Pharma	University	Federal
Reasons to participate			
Contribute to science and help others	*“I would like to know that I could possibly help with medical research and make someone potentially feel better.”*	*"I feel that a university is really trying to learn and put out good information... because they have no monetary reason to do it."*	*“People like me are a large component in helping researchers better understand conditions and diseases, so I would be willing to help.”*
Trust and credibility	“*It's a pharmaceutical company doing it so they seem trustworthy in that area”*	*“I would trust university sponsored research most of all; while being targeted in the Facebook content would give me pause, I would be able to acknowledge that it is an easy way to target a specific demographic. I would expect university researchers to have fewer issues with unethical practices.”*	*“I would be willing to consider participating in a government sponsored study because I would trust that it wouldn't be for profit or marketing.”*
Personal or familial experience with depression	*“I would be willing because I have a family history of depression and have seen some of it's detrimental effects first-hand.”*	*“I have been impacted by this ailment in the past which provides an incentive for me to help in research.”*	*“I would be interested in discussing my past connection to this condition. It would be an altruistic gesture of good knowledge about my past.”*
For payment	*“Pharmaceutical studies usually pay well and if the study helps me with a medical condition, that's also a benefit.”*	*“I would be willing to participate based on the monetary incentive.”*	*“It would likely pay very well and be professional structured.”*

© 2019 Pratap A et al. *JAMA Network Open.*

Reasons to not participate			
Privacy/data security concerns	*“I wouldn't want to participate in a study for a large company because I don't fully trust them to keep my information private.”*	*“It would feel a little bit too private and personal.”*	*“I prefer not to contribute information to the federal government database because I do not trust that the government will solely use that information for those purposes only”*
Mistrust or lack of credibility	*“I do not trust nor do I respect pharmaceutical companies. I believe there only interest is profit for themselves and not in the best interests of the public in general.”*	*“I would not want to participate in a study that was not conducted by a university with a proven track record”*	*“I don't trust the government's motive behind the research.”*

© 2019 Pratap A et al. *JAMA Network Open.*

eTable 4: Reasons for agreeing or declining to share social media data for research studies

	Pharma	University	Federal
**Reasons to share**			
Contribute to science and help others	*“because I think that even though the company is profit oriented, by sharing my posts and history maybe the company will be able to help someone upon studying my results and applying them to others.”*	*“If it helped further research on something and helped people out, I probably would.”*	*“Once again, I would like to help people with depression and look for alternative solutions.”*
Trust and credibility	--	*“I would have more trust in the management, security, and valuing of my data with a university, which I would believe to be more motivated by knowledge than profits.”*	*“They can be trusted and would likely keep all information confidential. The employees also likely have federal clearances.”*
For payment	*“I would be willing, but I think the compensation would have to be really high. I'm iffy about sharing my social media data for health related reasons.”*	*“I would be interested in earning money in my free time helping research.”*	*“I would share my Twitter data for the right amount of money.”*

© 2019 Pratap A et al. *JAMA Network Open.*

**Reasons not to share**			
Privacy/data security concerns	*“I would not want to share my social media information with a pharmaceutical company for a research study because I wouldn't trust the company to keep my data private and safe. I would worry about having my personal information marketed to third parties.”*	*“I don't care who has your data, there is NO guarantee that it will not be shared or hacked.”*	*“Our privacy is invaded enough as it is by the government. I wouldn't willingly hand social media data to anything federally sponsored."*
Mistrust or lack of credibility	*"I don't trust pharmaceutical companies with my private data. They are primarily interested in profits, ethics be damned."*	--	*"I think they will be sneaky and not fully inform me of how my data is being used."*
News of recent data breaches	*“With the big thing that went on with Facebook user's information being compromised months ago, I don't feel comfortable sharing my data with anyone.”*	*“I just don't share any social media data especially after the Cambridge Analytica fiasco.”*	*“Probably not, unless it was completely anonymous and Facebook has had some real privacy issues lately.”*

© 2019 Pratap A et al. *JAMA Network Open.*
